# Cone-Beam Computed Tomography and Radiographs in Dentistry: Aspects Related to Radiation Dose

**DOI:** 10.1155/2012/813768

**Published:** 2012-04-04

**Authors:** Diego Coelho Lorenzoni, Ana Maria Bolognese, Daniela Gamba Garib, Fabio Ribeiro Guedes, Eduardo Franzotti Sant'Anna

**Affiliations:** ^1^Department of Orthodontics, Federal University of Rio de Janeiro Dental School, Avenida Professor Rodolpho de Paulo Rocco, Ilha do Fundão 21941-590, Rio de Janeiro, RJ, Brazil; ^2^Department of Orthodontics, Bauru Dental School and Hospital of Rehabilitation of Craniofacial Anomalies, Universtity of São Paulo, 17012-101 Bauru, SP, Brazil; ^3^Department of Dental Radiology, Federal University of Rio de Janeiro Dental School, 21941-590 Rio de Janeiro, RJ, Brazil

## Abstract

*Introduction*. The aim of this study was to discuss the radiation doses associated with plain radiographs, cone-beam computed tomography (CBCT), and conventional computed tomography (CT) in dentistry, with a special focus on orthodontics. *Methods*. A systematic search for articles was realized by MEDLINE from 1997–March 2011. *Results*. Twenty-seven articles met the established criteria. The data of these papers were grouped in a table and discussed. *Conclusions*. Increases in kV, mA, exposure time, and field of view (FOV) increase the radiation dose. The dose for CT is greater than other modalities. When the full-mouth series (FMX) is performed with round collimation, the orthodontic radiographs transmit higher dose than most of the large FOV CBCT, but it can be reduced if used rectangular collimation, showing lower effective dose than large FOV CBCT. Despite the image quality, the CBCT does not replace the FMX. In addition to the radiation dose, image quality and diagnostic needs should be strongly taken into account.

## 1. Introduction

The high prevalence and increase in the number of children receiving orthodontic care [1] bring up an important issue: the use of ionizing radiation for diagnosis also increases the potential impact on public health [2]. These concerns exist because of the ability of X-rays to induce mutations in DNA, thereby increasing the risk of cancer [3]. Moreover, children may express increased susceptibility to environmental hazards, chronic infection and inflammation, dietary factors, and long-term medication due to differences in the uptake, metabolism, and excretion of potential mutagens [4] and a recent study has suggested a relationship between exposure to dental radiographs and a greater risk of thyroid cancer [5].

During the last century, dental diagnostic imaging was dominated by radiographs, which are two-dimensional representations of three-dimensional structures, with associated overlap and distortion. With the introduction of cone-beam computed tomography (CBCT), there was much interest in the technology due to its advantages: improved image quality, three-dimensional reconstruction, a 1 : 1 ratio that allowed reliable measurements, the possibility for craniofacial visualization, and lower radiation doses compared to traditional CT.

 However, it is necessary to monitor the radiation doses involved in these exams. Some concepts are relevant for this understanding, such as the methodology employed in research studies within the field. The majority of these studies use human head and neck phantoms built with tissues that mimic human tissues in regard to layers and radiation absorption. In some models, human skeletons are used [6]. The phantom is made in the form of detachable cross-sections with apertures created for the placement of dosimeters in the regions of interest. Many of these locations would be unfeasible *in vivo*. The dosimeters measure the absorbed dose in each region/tissue.

 The description of the radiation dose transmitted to the patient must be based on the effective dose (*E*), measured in Sieverts (Sv). This description is recommended by the International Commission on Radiological Protection (ICRP) [7] because it considers not only the dose, but also the type, quantity, sensitivity, and carcinogenic potential of the irradiated tissue [8]. Current estimates of per capita annual U.S. dose are 6200 *μ*Sv with almost 3000 *μ*Sv coming from diagnostic procedures. Ubiquitous background sources account for 3100 *μ*Sv annual dose or 8.5 *μ*Sv per day [9].

The effective dose in a given tissue (*E*_*T*_) is calculated by the following equation [10]: *E*_*T*_ = *w*_*T*_ · *H*_*T*_, where *w*_*T*_ is the tissue weighting factor, which represents the radiosensitivity of the tissue/organ and thereby the contribution of this tissue to the total risk, and *H*_*T*_ is the equivalent dose for each tissue/organ. The sum (∑) of the *E*_*T*_ for each tissue/organ provides the total effective dose (*E*).

 The equivalent dose (*H*_*T*_) for a tissue/organ, in Sv, is represented by the following formula: *H*_*T*_ = *w*_*R*_ · *D*_*T*_ · *f*_*T*_, where *w*_*R*_ is the radiation weighting factor (for X-rays, this value is 1), *D*_*T*_ is the mean dose absorbed in the dosimeters in gray (Gy), and *f*_*T*_ is the irradiated fraction of tissue in relation to its total volume in the body (normal values described in the literature) [11].

The tissue/organ weighting factors, w_T,_ are provided and updated by the ICRP (Table 1). The most widely used version is from 1990 [7] and is based on mortality rates used to estimate the risk of cancers in various tissues. Updates in 2005 [12] and 2007 [10] included the salivary glands and changes in some tissue-weighting factors according to recent rates of cancer incidence, which are better descriptors of cancer burden, especially for those cancer types with high survival rates [13]. The recommendations from 2005 were the draft for the ICRP 2007 recommendations, and the two are, therefore, relatively comparable. Thus, depending on the version of the ICRP recommendations, different effective doses are found for the same level of irradiation. Some articles use the absorbed dose (Gy), which is less relevant because it does not consider the relative contribution of different organs/tissues to the total risk [14].

When using ionizing radiation, the ALARA [15] (as low as reasonably achievable) principle must be respected. Nevertheless, discussions about radiation doses and their contributing factors do exist, and this requires vigilance in obtaining the best possible cost-benefit relationship between dosage and information. Consequently, the sources of radiation used in dentistry (radiography, CBCT, and CT) and the influence of the image acquisition protocol on these doses is discussed, especially in orthodontics.

## 2. Materials and Methods

### 2.1. Literature Search Strategy

**Figure 1:**
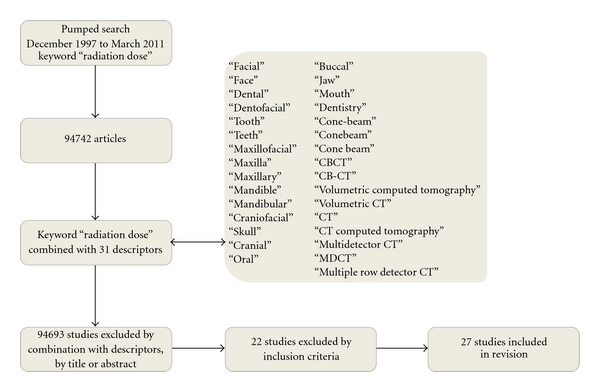
Flow chart of the search process.

The literature on radiation doses used in dentistry was systematically reviewed. The articles were located by an online search using MEDLINE from 1997 to March 2011. The keyword used in this search was “radiation dose,” combined with 31 descriptors to restrict it to dentistry (Figure 1). The bibliographies of the selected articles were analyzed in search of research that was not found on MEDLINE. 

### 2.2. Inclusion Criteria for Articles

 Initially, articles in English were selected according to their title and abstract, followed by a complete reading of the text. The studies included in the analysis fulfilled the following criteria:

1. evaluation of radiation dose in radiographs and/or CBCT and/or CTs used in dentistry;
2. the use of phantom or thermoluminescent dosimeters;
3. results that showed effective dose and ICRP used;
4. tomography of the maxilla and/or mandible and/or the entire head with the assessments of smaller areas discarded;
5. radiographs included, including a complete periapical examination, and/or a complete interproximal examination, and/or a panoramic and/or lateral cephalometric/PA and/or maxillary/mandibular occlusal examination.

The CBCT studies were divided according to their FOV [11]: *small FOV* (spherical diameter or cylinder height ≤10 cm; captures most of one or both arches, but not all of the anatomy of the maxilla); *medium FOV* (spherical diameter or cylinder height between 10 and 15 cm; captures the entire dentition and temporomandibular joints, but generally does not include the complete soft profile of the chin and nose, which is necessary for orthodontic care); *large or extended FOV* (spherical diameter or cylinder height >15 cm; captures the maxillofacial complex, chin and nose).

## 3. Results

 There were 94.742 articles identified with the keyword *radiation dose*, which were reduced to 27 after application of the criteria.  Table 2 lists these data. It is important to know that some of the devices presented in Table 2 are not the most current versions available. For example, the CBCT devices such as Classic i-CAT, NewTom 9000, NewTom 3G, and Iluma already have new versions (Next Generation i-CAT, NewYom 5G and Iluma Elite). The CB MercuRay is not currently available for purchase. They were all kept in Table 2 because they can still be used in some centers.

## 4. Discussion

Methodological variations explain the different doses for the same exam, where these include phantoms made by different companies or positioned asymmetrically, as well as variations in dosimeters, their sensors [16], their locations on the phantoms, and their number [17]. Many researchers do not include the calvaria [6, 8, 15, 18–24] and cervical vertebrae [18, 21, 23, 24] when counting the red bone marrow, esophagus [8, 18–21, 23–26], skin [25], and remaining tissues in the calculation of the effective dose [6, 18, 19, 21, 23, 24]. The ICRP version used is important due to the inherent variations in the different weighting factors. The 1990 ICRP [7] did not include the salivary glands, which are highly irradiated in dentistry, and some authors included them among the remainder tissues of the ICRP, which considerably increased the effective dose (Table 2). This tissue was incorporated in the ICRP from 2005 [12] and 2007 [10], and this explains the larger doses measured.

### 4.1. Image Acquisition Protocol

 Increases in kV, mA, and exposure time result in higher effective doses for any exam [6, 11, 12, 16, 27–29]. The adjustments in CBCT images vary; for the i-CAT, the kV, mA, and exposure time are established by the manufacturer and do not vary from patient to patient. That is, the same dose is used for patients of different sizes and different ages. In children, this may be higher than needed for a diagnosis. For the NewTom 3G, exposure is also set by the manufacturer, but a dynamic process identifies the radiation needed, and the mA is adjusted during the exposure. For the CB MercuRay, the operator defines kV and mA. Inexperienced operators tend to increase kV and mA because the overexposed images appear to be adequate with reduced noise, which increases the risk of overexposure [12].

For CBCT, smaller FOV normally generates lower radiation doses, similar to the action of collimators [6, 12, 17, 27–29]. In general, the mandibular FOV has a larger dose than the maxillary [27, 30], because the salivary glands, thyroid, and esophagus are more irradiated in this exam. The chosen FOV must be the smallest that will encompass the region of interest [6]. For example, the medium FOV (13 cm) from the NewTom/i-CAT is often enough to reach the regions required in many children for orthodontics. With the large FOV, unnecessary areas are irradiated in these “minor” children, increasing the effective dose. On the other hand, the large FOV is always necessary in adults. The operator is responsible for choosing the appropriate FOV, large or medium, according to the size of the child.

### 4.2. CBCT *versus* CT

 The effective dose generated by CT is generally higher than that of CBCT. When analyzing the dose according the 2007 ICRP, the head CT requires doses between 995 and 1160 *μ*Sv, whereas the large FOV CBCT requires 30 to 68 *μ*Sv for the NewTom 3G, 74 *μ*Sv for the Next Generation i-CAT, 82 to 182.1 *μ*Sv for the Classic i-CAT, 87 *μ*Sv for the SkyView, 93 to 260 *μ*Sv for the Kodak 9500, and 98 to 498 *μ*Sv for the Iluma. The CB MercuRay approaches the radiation levels of standard CT, with doses between 569 and 1073 *μ*Sv. High doses are observed for CT even when areas are reduced, ranging between 534 and 860 *μ*Sv for the maxilla and mandible. This represents a higher dose emitted by CT, especially in relation to the NewTom 3G and i-CAT CBCT devices. The CT dose is also high in relation to radiographs, which emit doses of 14.2 to 24.3 *μ*Sv for the panoramic radiograph, 5.4 *μ*Sv for the lateral cephalometric radiograph and 34.9 to 170.7 *μ*Sv for a complete intraoral examination.

### 4.3. CBCT *versus* Conventional Radiographs

 In this transition phase of image diagnosis, a question frequently arises: “to how many radiographs is the radiation dose of CBCT equivalent?” Despite the straightforward nature of the question, the answer involves many nuances.

The characteristics of an intraoral radiograph influence its effective dose, such as film sensitivity (when not digital) and, especially, the type of collimation (rectangular or circular). Intraoral radiographs with circular collimation and films that are not sensitive (D-speed) yielding doses that are much greater than sensitive (E/F-speed) and digital films with rectangular collimation. The dose for the digital/F-speed complete intraoral exam with rectangular collimation (34.9 *μ*Sv) is close to 4.9 times lower than one with circular collimation (170.7 *μ*Sv) [13]. The NCRP [31] and the American Dental Association [32] recommend rectangular collimation for periapical and bitewing radiographs, the use of a thyroid protector and the avoidance of using films lower than E-speed (preferably F-speed/digital). In terms of extraoral radiographs, according to ICRP 2005/2007, the doses are between 2.7 and 24.3 *μ*Sv for the panoramic and 5.6 *μ*Sv for the lateral cephalometric.

Many orthodontists do not request a full-mouth series of intraoral radiographs for orthodontic planning and this practice greatly reduces the dose of radiation imparted to the patient when compared to CBCT exposure. This is particularly important when dealing with young children that are more susceptible to radiation [4]. However, in some instances, it hampers the diagnosis since the panoramic radiograph shows large distortions that prevent the diagnosis of more subtle changes, such as caries and root resorption in early stages. Thus, these radiographs should be taken in patients with permanent dentition that will begin full braces orthodontic treatment to search for dental diseases and to serve as a precise record of each teeth and adjacent bone during and posttreatment. Panoramics should also be taken during comprehensive orthodontic treatment to visualize the entire maxilla and mandible including the teeth, maxillary sinuses, nasal cavity, and condyles.

Therefore, in the initial orthodontic radiographic documentation (ORD), which often includes full mouth series of intraoral radiographs (FMX), panoramic, and lateral cephalometric radiographs, the total dose varies between 43.2 and 200.6 *μ*Sv, depending on the collimation of intraoral radiographs. The large FOV of most CBCT scanners provides lower doses than the ORD with FMX using circular collimation. If rectangular collimation is used, the ORD presents lower effective dose.

It is not enough to compare doses between diagnostic procedures, because diagnostic quality cannot be separated from the dose used. Objective studies of the impact of CBCT image quality on diagnostic performance must be conducted before any definitive conclusions are drawn about the differences generated by reduced doses [12]. Current data describe the reconstructions of lateral teleradiography of CBCT as having similar precision to conventional radiographs [33] in addition to high intra- and interexaminer reproducibility [34]. Comparisons between CBCT images, periapical radiography, and clinical evaluations have not demonstrated significant differences in the extent of periodontal defects, but CBCT allows for the observation of all bone defects and better inspection of craters and furcation defects [35]. However, delicate structures such as the trabecular bone and the periodontal ligament display lower visibility and higher variability between CBCT and CT than do other structures [36]. Conventional radiography has advantages in terms of contrast, the quality of the bone image and delineation of the lamina dura, in addition to superior performance in the evaluation of the periodontal space compared to CBCT [37] and is, therefore, indispensable for accurate periapical diagnosis.

### 4.4. Differences between CBCT Devices

The CBCT dose varies according to the CBCT device. Among the better known large FOV CBCT, the CB MercuRay provides the greatest radiation, followed by the Classic i-CAT, the Kodak 9500, the Iluma, the Next Generation i-CAT, and the NewTom 3G. Considering the large FOV (ICRP2005) [12], the radiation doses of the Classic i-CAT and the CB MercuRay are 3.3 and 9.5 to 17 times greater, respectively, than that of the NewTom 3G. The Next Generation i-CAT comes close to the NewTom 3G (ICRP 2007) in terms of radiation level because it scans more quickly than the Classic i-CAT.

Considering the large FOV CBCT, a general conclusion, based on values described in Table 2, is that the effective doses from most devices are found in the 30–200 *μ*Sv range. Although the geometry of image acquisition is basically the same, the differences in collimation of the cone beam, as well as the X-ray exposure factors, lead to considerable differences in absorbed dose for all organs in the head and the neck regions. A single effective dose is not a concept that should be used for CBCT when compared to alternative radiographic methods such as panoramic, intraoral radiography, and conventional CT. The range of doses among devices is too large to consider them as a single modality [17].

In addition to controlling the settings of tomographs, radiation levels can vary due to exposure times and radiation beams. The NewTom 3G scans in 36 s but emits X-ray for only 5.4 s. Similarly, the Classic i-CAT (FOV 13 cm) scans in 20 s, but the X-ray tube is only activated for 3.3 s. The large FOV in the i-CAT involves two FOV 13 cm scans, performed sequentially and interlaced to create a greater volume. Double scanning preserves the quality of FOV 13 cm but requires almost double the exposure time. The CB MercuRay scans in 11 s and emits for 10 s. Thus, the exposure for the CB MercuRay is continuous, whereas for the NewTom 3G and the i-CAT it is pulsed; consequently, the latter two use radiation more efficiently because the detector is only exposed while it registers photons and because radiation is not emitted while the detector transfers the image signal to the computer [12].

The results of the CBCT devices expressed in Table 2 should be interpreted carefully due to the interplay among image quality, the size of the scanned volume, and the absorbed radiation dose in different tissues. Comparisons of the performances of CBCT devices cannot be done based on dosimetric results alone. The radiation dose from these devices can be seen as a function of the diagnostic application. The two key factors for an acceptable image are an appropriate size and positioning of the FOV and an acceptable quality of the reconstructed image [17], a point that was not evaluated in this revision. Further study is required to bring the image quality into play, on a technical and diagnostic level. By investigating technical image quality, the relation between the exposure from CBCT devices and the image quality performance can be quantified [17].

## 5. Conclusions

1. Increases in kV, mA, exposure time, and FOV increase the dose of radiation, regardless of the type of exam.
2. The effective dose for CT is greater than for CBCT or conventional radiographs.
3. When the FMX is performed with round collimation, the ORD issues higher doses than most of the large FOV CBCT. Radiation dose for ORD can be lower than large FOV CBCT if rectangular collimation is used in FMX. Despite the image quality, CBCT does not replace the FMX and most orthodontic cases will be properly handled with conventional 2D radiographs. CBCT should be required for more complex cases.
4. The orthodontists have the duty to preserve the health of the patient and always seek the best treatment. This quest begins with exams that require the least amount of radiation dose to treat the patient appropriately.

## Figures and Tables

**Table 1:** Tissue-weighting factors for calculation of effective radiation dose.

Tissue	ICRP 1990	ICRP 2005	ICRP 2007
*Bone Marrow*	0.12	0.12	0.12
Breast	0.05	0.12	0.12
Colon	0.12	0.12	0.12
Lung	0.12	0.12	0.12
Stomach	0.12	0.12	0.12
Gonads	0.20	0.05	0.08
*Esophagus*	0.05	0.05	0.04
Bladder	0.05	0.05	0.04
Liver	0.05	0.05	0.04
*Thyroid*	0.05	0.05	0.04
*Bone surface*	0.01	0.01	0.01
*Brain*	RT	0.01	0.01
*Skin*	0.01	0.01	0.01
*Salivary glands*	Not included	0.01	0.01
Kidney	RT	0.01	RT
*Remainder Tissues*	0.05^a^	0.10^b^	0.12^c^

RT: Remainder tissues; ^a^adrenals/*brain*/upper large intestine/small intestine/kidney/*muscle*/pancreas/spleen/thymus/uterus

^b^Adipose tissue/adrenals/connective tissue/*extrathoracic airways*/gallbladder/heart wall/*lymphatic nodes/muscle*/pancreas/prostate/spleen/thymus/uterus/cervix

^c^Adrenals/*extrathoracic region*/gallbladder/heart/prostate/kidneys/small intestine/*lymphatic nodes*/*oral mucosa/muscle*/pancreas/spleen/thymus/uterus/cervix (text in *boldface* represents tissues used for calculation of maxillofacial dose).

**Table 2:** Effective doses. (ExcGland or IncGland: salivary glands excluded or included; Mx: Maxilla; Md: Mandible).

Exams/equipment/adjustment provided	Effective Dose (*μ*Sv)			
	ICRP 60-1990		ICRP 2005	ICRP 103-2007
	ExcGland	IncGland		
PANORAMIC RADIOGRAPHS				

PM2002CCProlinePlanmeca/70 kVp/7 mA/18 s [25]	3.8			
VeraviewepocsMorita 77 kV/5 mA/8.1 s [8]			5.2	
OrthophosSiemens/62 kV/16 mA/14.1 s [38]	9	16.4		
PM2002CCProLinePlanmeca/64 kV/6 mA/15 s [39]	4	9		
PromaxPlanmeca/66 kV/6 mA/16 s [16]	17	26		
PM2002CCProlinePlanmeca/73 kV/5 mA/15 s [18]		10		
Digital/PM2002CCProline2000Planmeca/66 kV/4 mA/18 s [16]	8	12		
Digital/PM2002CCProline2000Planmeca/66 kV/8 mA/18 s [16]	23	38		
Digital/CranexExcelSoredex/65 kV/6 mA/19 s [40]	4.5	12.3		
Digital/Verawiewepocs5DMorita/70 kV/4 mA/8.2 s [40]	2.5	5.5		
Digital/ECProlinePlanmeca/64 kV/7 mA/18.3 s [40]	5.7	14.9		
Digital/Orthoralix9200DDEGendex/74 kV/4 mA/12 s [40]	2.4	4.7		
Digital/ProMaxPlanmeca/Adult [6]	20		23	
Digital/ProMaxPlanmeca/68 kV/13 mA/16 s [13]	7.1			24.3
Digital/OrthophosXGSirona/64 kV/8 mA/14.1 s [13]	4.3			14.2
Digital/OrthophosPlusDSSirona/66 kVp/16 mA/14.1 s [30]	6.2	22		
Digital/VeraviewepocsMorita/67 kV/5 mA/8.1 s [8]			2.7	
Digital/Veraviewepocs3DMorita/70 kV/5 mA/7.4 s [8]			2.9	
Digital/CranexTomeSoredex/70 kV/4 mA/15 s [40]	3.3	8.1		

LATERAL CEPHALOMETRIC RADIOGRAPHS				

OrthophosCSiemens/77 kV/14 mA/0.5 s [19]	2.3			
PM2002CCProLinePlanmeca/70 kV/12 mA/0.9 s [39]	2	3		
CranexTomeSoredex/70 kVp/10 mA/0.4 s [20]	3	3.7		
CranexTomeSoredex/Collimation/70 kVp/10 mA/0.4 s [20]	1.6	2.2		
PM2002CCProlinePlanmeca/80 kV/12 mA/0.5 s [18]		5		
Digital/OrthophosDSCephSiemens/73 kV/15 mA/15.8 s [19]	1.1			
Digital/ProLineCephCMPlanmeca/Collimation/70 kVp/10 mA/23 s [21]	1.7	3.4		
Digital/CranexTomeSoredex/Collimation/70 kVp/4 mAs [21]	1.6	2.2		
Digital/InterayVarian/77 kVp/6.5 mAs [13]	3.7			5.6

PA CEPHALOMETRIC RADIOGRAPHS				

Digital/InterayVarian/75 kVp/11 mAs [13]	3.9			5.1

INTRAORAL RADIOGRAPHS				
IntraPlanmeca/FullMouthRadiographs/70 kV/8 mA/Digital or F-speed film/RectangularCollimation [13]	12.2			34.9
IntraPlanmeca/FullMouthRadiographs/70 kV/8 mA/Digital or F-speed film/RoundCollimation [13]	58.4			170.7
IntraPlanmeca/FullMouthRadiographs/RoundCollimation/Adult [6]	115		129	
IntraPlanmeca/Bitewing(04)/70 kV/8 mA/Digital or F-speed film/RectangularCollimation [6]	1			5
SiemensHeliodent70Dentotime/OcclusalMx [18]		7		

LARGE FOV CONE BEAM CT				

Classic i-CAT/FOV22 cm/120 kV/3–8 mA [27]	92.8			182.1
Classic i-CAT/FOV22 cm/120 kV/5.7 mA [12]	134.8		193.4	
Classic i-CAT/FOV22 cm/120 kV/3–8 mA/2 × 20 s [28]				82
Next Generation i-CAT/FOV23 cm/120 kV/5 mA/19 mAs/8.9 s [11]	37			74
NewTom3G/FOV19 cm/110 kV/1.5 mA/8.09 mAs/36 s [11, 12]	44.7		58.9	68
NewTom3G/FOV19 cm/110 kV/<15 mA [28]				30
NewTom9000/FOV23 cm/110 kV/5.4 mA [15]			56.2	
CBMercuRay/FOV19 cm/100 kV/10 mA/100 mAs/10 s [11, 12]	476.6		557.6	569
CBMercuRay/FOV19 cm/120 kV/15 mA/150 mAs/10 s [11, 12]	846.9		1025.4	1073
CBMercuRay/FOV19 cm/100 kV/15 mA [6]	415		479	
CBMercuRay/FOV19 cm/120 kV/15 mA [6]	656		761	
CBMercuRay/FOV19 cm/100 kV/10 mA [6]	264		306	
CBMercuRay/FOV19 cm/100 kV/5 mA [6]	153		177	
CBMercuRay/FOV19 cm/100 kV/2 mA [6]	75		86	
Iluma/FOV19 cm/120 kV/1 mA/20 mAs/20 s [11]	50			98
Iluma/FOV19 cm/120 kV/3.8 mA/152 mAs/40 s [11]	252			498
Kodak9500/FOV18 cm/80 kV/86.4 mAs [29]	52			93
Kodak9500/FOV18 cm/85 kV/108 mAs [29]	92			163
Kodak9500/FOV18 cm/90 kV/108 mAs [29]	148			260
Kodak9500/FOV18 cm/90 kV/108 mAs [17]				136
SkyView/FOV17 cm/90 kV/51 mAs [17]				87

MEDIUM FOV CONE BEAM CT				

Classic i-CAT/FOV13 cm/120 kV/3–8 mA [27]	39.5			110.5
Classic i-CAT/FOV13 cm/120 kV/5.7 mA [12]	68.7		104.5	
Classic i-CAT/FOV13 cm/120 kV/23.87 mA [15]			61.1	
Classic i-CAT/FOV13 cm/120 kV/3–8 mA/10 s [28]				48
Classic i-CAT/FOV13 cm/120 kV/3–8 mA/40 s [28]				77
Classic i-CAT/FOV13 cm/120 kV/5 mA/19 mAs/20 s [11]	29			69
Next Generation i-CAT/FOV13 cm/120 kV/5 mA/19 mAs/8.9 s [11]	36			87
Next Generation i-CAT/FOV13 cm/120 kV/18.5 mAs [17]				83
NewTom9000/FOV13 cm/110 kV/3.2 mA [30]	36.9	77.9		
NewTom9000/FOV13 cm/110 kV/3.5 mA/18 s [26]	50.3			
NewTom9000/FOV13 cm/110 kV/3.4 mA/17 s [22]	35	64		
NewTom9000/FOV13 cm/110 kV/3.4 mA/17 s/Thyroid Protector [22]	23	52		
NewTom3G/FOV15 cm/110 kV/<15 mA [28]				57
NewTom5Gi/FOV15 cm/110 kV/8.8 mAs [17]				194
CBMercuRay/FOV15 cm/120 kV/15 mA/120/mAs/10 s [11]	288.9		435.5	560
CBMercuRay/FOV15 cm/100 kV/15 mA [6]	354		402	
CBMercuRay/FOV15 cm/120 kV/15 mA [6]	601		680	
Galileos/FOV15 cm/85 kV/5 mA/21 mAs/14 s [11]	28			70
Galileos/FOV15 cm/85 kV/7 mA/42 mAs/14 s [11]	52			128
GalileosComfort/FOV15 cm/85 kV/28 mAs [17]				84
Kodak9500/FOV15 cm/80 kV/86.4 mAs [29]	39			76
Kodak9500/FOV15 cm/85 kV/108 mAs [29]	49			98
Kodak9500/FOV15 cm/90 kv/108 mAs [29]	76			166
IlumaElite/FOV14 cm/120 kV/76 mAs [17]				368
Scanora3D/FOV13.5 cm/85 kV/48 mAs [17]				68

SMALL FOV CONE BEAM CT				

Classic i-CAT/FOV6 cmMx/120 kV/3–8 mA [27]	9.7			36.5
Classic i-CAT/FOV6 cmMx/120 kV/3–8 mA/HighResolution [27]	18.5			68.3
Classic i-CAT/FOV6 cmMx/120 kV/3–8 mA/20 s [28]				45
Classic i-CAT/FOV6 cmMx/120 kV/3–8 mA/40 s [28]				77
Classic i-CAT/FOV6 cmMd/120 kV/3–8 mA [27]	23.9			75.3
Classic i-CAT/FOV6 cmMd/120 kV/3–8 mA/HighResolution [27]	47.2			148.5
Classic i-CAT/FOV6 cmMd/120 kV/3–8 mA/20 s [28]				34
Classic i-CAT/FOV6 cmMd/120 kV/3–8 mA/40 s [28]				64
Classic i-CAT/FOV8 cm/120 kV/3–8 mA/40 s [28]				37
Next Generation i-CAT/FOV6 cmMd/120 kV/18.5 mAs [17]				45
NewTom9000/FOVMx [30]	19.9	41.5		
NewTom9000/FOVMd [30]	34.7	74.7		
NewTom5G/FOV10 cm/110 kV/10.4 mAs [17]				83
NewTom5Gi/FOV8 cm/110 kV/43 mAs [17]				265
CBMercuRay/FOV10 cmMx/120 kV/15 mA/150 mAs/10 s [11, 12]	168.4		283.3	407
CBMercuRay/FOV10 cm/100 kV/15 mA [6]	328		369	
CBMercuRay/FOV10 cm/120 kV/15 mA [6]	535		603	
CBMercuRay/FOV10 cm/120 kV/15 mA [23]	451.8			510.5
Promax3D/FOV8 cm/84 kVp/12 mA/6 s [41]	269			674
Promax3D/FOV8 cm/84 kV/12 mA/72 mAs/18 s [11]	151			488
Promax3D/FOV8 cm/84 kV/16 mA/96 mAs/18 s [11]	203			652
Promax3D/FOV8 cm/84 kV/8 mA/12 s/NormalResolution [42]				102
Promax3D/FOV8 cm/84 kV/10 mA/12 s/NormalResolution [42]				169
Promax3D/FOV8 cm/84 kV/12 mA/12 s/NormalResolution [42]				216
Promax3D/FOV8 cm/84 kV/14 mA/12 s/NormalResolution [42]				272
Promax3D/FOV8 cm/84 kV/16 mA/12 s/NormalResolution [42]				298
Promax3D/FOV8 cm/84 kV/8 mA/2.8 s/LowDose [42]				30
Promax3D/FOV8 cm/84 kV/16 mA/12 s/HighDose [42]				306
Promax3D/FOV8 cm/84 kV/8 mA/8.3 s/LowDose [42]				87
Promax3D/FOV8 cm/84 kV/169 mAs/HighDose [17]				122
Promax3D/FOV8 cm/84 kV/19.9 mAs/LowDose [17]				28
PreXion3D/FOV8.1 cm/90 kV/4 mA/76 mAs/19 s [11]	66			189
PreXion3D/FOV8.1 cm/90 kV/4 mA/148 mAs/37 s [11]	154			388
3D Accuitomo 170/FOV5 cmMx/90 kV/87.5 mAs [17]				54
Kodak9500/FOV8 cm/90 kV/108 mAs [17]				92
PicassoTrio HighDose/FOV7 cm/85 kV/127 mAs [17]				123
PicassoTrio LowDose/FOV7 cm/85 kV/91 mAs [17]				81
Scanora 3D/FOV7.5 cmMx/85 kV/30 mAs [17]				46
Scanora 3D/FOV7.5 cmMd/85 kV/30 mAs [17]				47
Scanora 3D/FOV7.5 cmMxMd/85 kV/30 mAs [17]				45
Veraviewpocs3D/FOV8 cm/70 kV/51 mAs [17]				73

CONVENTIONAL CT				

SomatomVolumeZoom4/Scan22.6 cmFullHead/120 kV/ 90 mA/44.12 s/Slice0.75 mm [28]				1110
SomatomSensation16/Scan22.5 cmFullHeadl/120 kV/90 mA/29.48 s/slice0.75 mm [28]				995
Mx8000IDTPhilips/Scan22.5 cmFullHead/120 kV/140 mA/29.6 s/Slice0.75 mm [28]				1160
Somatom64/Scan12 cm/120 kV/90 mA [11]	453			860
Somatom64CareDose4D/Scan12 cm/120 kV/90 mA [11]	285			534
SomatomPlusVolumeZoom4/ScanMx+Md/Slice1.25 mm/21.25 s/120 kVp/150 mA [18]		2110		
SomatomSensation/Scan10 cm/120 kV/90 mA [15]			429.7	
ExcelTwin/Scan9.6 cm/120 kV/300 mAs/Slice2 mm/2sporslice [39]	314	924		
HiSpeedQX/i/Scan7.7 cmMx+Md/120 kV/100 mA [23]	595.6			768.9
SomatomVolumeZoom4/Scan7.2 cmMd/120 kV/90 mA/15.16 s/Slice0.75 mm [28]				494
SomatomSensation16/Scan6.3 cmMd/120 kV/90 mA/7.87 s/Slice0.75 m [28]				474
Mx8000IDTPhilips/Scan6 cmMd/120 kV/140 mA/7.89 s/Slice0.75 mm [28]				541
SomatomPlus4VolumeZoom Scan5.2 cmMd/120 kV/100 mAs [24]			250	
ElscintExcel2400/ScanMd/120 kVp/315 mAs [43]	2426	3324		
SomatomPlus4VolumeZoom/ScanMd/Slice1.25 mm/12.64 s/120 kVp/150 mA [18]		1320		
SomatomPlusVolumeZoom4/ScanMx/Slice1.25 mm/9.47 s/ 120 kVp/150 mA [18]		1400		
ElscintExcel2400/ScanMx/120 kVp/315 mAs [43]	1031	1202		
